# Associations of wheezing phenotypes with late asthma outcomes in the Avon Longitudinal Study of Parents and Children: A population-based birth cohort

**DOI:** 10.1016/j.jaci.2016.01.046

**Published:** 2016-10

**Authors:** Raquel Granell, A. John Henderson, Jonathan A. Sterne

**Affiliations:** School of Social and Community Medicine, University of Bristol, Bristol, United Kingdom

**Keywords:** ALSPAC, wheezing phenotypes, childhood, adolescence, latent class, ALSPAC, Avon Longitudinal Study of Parents and Children, BDR, Bronchodilator reversibility, FEF_25-75_, Forced expiratory flow between 25% and 75%, FVC, Forced vital capacity, mOR, Multinomial odds ratio, OR, Odds ratio, SDU, SD units

## Abstract

**Background:**

Variable patterns of childhood wheezing might indicate differences in the cause and prognosis of respiratory illnesses. Better understanding of these patterns could facilitate identification of modifiable factors related to development of asthma.

**Objectives:**

We characterized childhood wheezing phenotypes from infancy to adolescence and their associations with asthma outcomes.

**Methods:**

Latent class analysis was used to derive phenotypes based on patterns of wheezing recorded at up to 14 time points from birth to 16½ years among 12,303 participants from the Avon Longitudinal Study of Parents and Children. Measures of lung function (FEV_1_, forced vital capacity [FVC], and forced expiratory flow between 25% and 75% [FEF_25-75_]) and fraction of exhaled nitric oxide (Feno) were made at 14 to 15 years of age.

**Results:**

Six wheezing phenotypes were identified: never/infrequent, preschool-onset remitting, midchildhood-onset remitting, school age–onset persisting, late childhood–onset persisting, and continuous wheeze. The 3 persistent phenotypes were associated with bronchodilator reversibility of 12% or greater (BDR) from baseline (odds ratio [OR] range, 2.14-3.34), a Feno value of 35 ppb or greater (OR range, 3.82-6.24), and lung function decrements (mean range of differences: −0.22 to −0.27 SD units (SDU) for FEV_1_/FVC ratio and −0.21 to −0.33 SDU for FEF_25-75_) compared with never/infrequent wheeze. Midchildhood-onset (4½ years) remitting wheeze was associated with BDR (OR, 1.77; 95% CI, 1.11-2.82), a Feno value of 35 ppb or greater (OR, 1.72; 95% CI, 1.14-2.59), FEV_1_/FVC ratio decrements (OR, −0.22 SDU; 95% CI, −0.36 to −0.08 SDU), and FEF_25-75_ decrements (OR, −0.16 SDU; 95% CI, −0.30 to −0.01 SDU). Preschool-onset (18 months) remitting wheeze was only associated with FEV_1_/FVC ratio decrements (OR, −0.15 SDU; 95% CI, −0.25 to −0.05 SDU) and FEF_25-75_ decrements (OR, −0.14 SDU; 95% CI, −0.24 to −0.04 SDU). The persisting phenotypes showed evidence of sex stratification during adolescence.

**Conclusions:**

Early childhood–onset wheezing that persists into adolescence represents the clearest target group for interventions to maximize lung function outcomes.

Asthma is a heterogeneous disease that displays phenotypic variation throughout the life course. Prospective longitudinal studies date the origins of a high proportion of asthma cases to early childhood,^1^ and variations in the natural history of childhood wheezing are associated with different long-term outcomes.^2^ Therefore the search for causal factors influencing the development and natural course of asthma has focused on early life. Cohort studies recruited in pregnancy or soon after birth have examined associations between early-life influences and childhood asthma, but only a few factors with modest effects have been identified, and these do not explain recent increases in childhood asthma prevalence. A possible explanation for difficulties in identifying causes of childhood wheezing is that phenotypic heterogeneity is a manifestation of underlying differences in the pathophysiology of asthma endotypes, each having different genetic and environmental influences. Characterization of wheezing phenotypes in children might help clarify the underlying mechanisms through which asthma occurs and improve the power to detect causal factors.

A seminal example of a temporal approach to characterizing wheezing phenotypes reported in 1995 by the Tucson Children's Respiratory Study^3^ drew attention to the distinction between transient early wheezing and persistent or later-onset wheezing in their associations with subsequent asthma history. Subsequently, we applied data-driven approaches to identify 6 wheezing phenotypes using repeated measures of wheezing in the Avon Longitudinal Study of Parents and Children (ALSPAC).^2^ Our methods and results have been replicated/validated in several independent cohorts, with remarkable similarity in the patterns of wheezing phenotypes characterized in different populations.^4^, ^5^ Wheezing phenotypes defined by using this approach have clear differences in their associations with clinical markers of asthma,^2^ and we have also reported associations with early risk factors^6^ and differences in genetic associations, suggesting that these are distinct biological entities.^7^, ^8^ Others have shown face validity of statistically derived phenotypes compared with those defined by using conventional clinical criteria and identified a novel clinical phenotype characterized by unremitting wheeze, low lung function, and poor bronchodilator response but without an asthma diagnosis.^9^ However, to date, there have been few novel insights into how modifiable environmental factors might influence the onset of specific asthma subtypes, which would be a prerequisite for primary or secondary prevention of disease.

Adolescence is a crucial period, as is very early childhood, in the natural history of asthma and wheezing illnesses. The most obvious manifestation is the well-described “sex switch” in asthma prevalence that occurs during the transition from early childhood to midchildhood, when male asthma predominates, to a female preponderance in young adults.^10^ There has been much speculation that hormonal influences, particularly the effect of estrogens on inflammation, could underpin this change by increasing asthma incidence in postpubertal female subjects.^11^ However, adolescence is a time of great changes in lifestyle and behaviors, as well as biological and physiologic processes. Therefore environmental risk factors during this period might influence asthma natural history and thus be amenable to modification.

The aims of this study were to define extended wheezing phenotypes by using repeat measurements of wheeze made regularly during the first 16½ years of life and to investigate associations of these extended phenotypes with physician-diagnosed asthma, lung function, and Feno measures at age 14 to 15 years.

## Methods

ALSPAC is a longitudinal, population-based birth cohort study that included 14,062 live-born children. The study protocol has been described previously,^12^ and further details can be found in the Methods section in this article's Online Repository at www.jacionline.org. Ethical approval for the study was obtained from the ALSPAC Ethics and Law Committee and Local Research Ethics Committees. The study Web site contains details of all the data that are available through a fully searchable data dictionary at www.bris.ac.uk/alspac/researchers/data-access/data-dictionary/.

### Asthma, atopy, and lung function outcomes

Doctor-diagnosed asthma ever was defined based on parental report at 91 months (7½ years) and 166 months (14 years). Based on parental report of doctor-diagnosed asthma ever at 91 months (7½ years) and 166 months (14 years) and parental report of current asthma at 166 months (14 years), we defined asthma status as follows: *no asthma* if the subject never had doctor-diagnosed asthma, *remittent* if doctor-diagnosed asthma was reported by 7½ years but no asthma symptoms were reported at 14 years, *incident* if doctor-diagnosed asthma was reported by 14 years but not at 7½ years, and *persistent* if doctor-diagnosed asthma was reported at 7½ and 14 years and asthma symptoms were reported at 14 years.

Atopy was measured at a research clinic at age 7½ years, as previously described.^13^

Spirometry was done in a research clinic at ages 8½ and 15 years by using methods described previously.^14^ Lung function at 15 years was defined as the highest of 3 measures before and 15 minutes after receiving 400 μg of salbutamol administered by using metered aerosol and a spacer.^15^, ^16^ Postsalbutamol lung function was used as the primary outcome of this study. Presalbutamol lung function measures were used for sensitivity analyses and for comparison with findings at 8 years. FEV_1_, forced vital capacity (FVC), and forced expiratory flow between 25% and 75% (FEF_25-75_) were converted into sex-, age-, and height-adjusted SD units (SDU).^17^, ^18^ From presalbutamol and postsalbutamol lung function measurements, we calculated percentage change of FEV_1_ from baseline as follows:

([Post-FEV_1_] − [Pre-FEV_1_])/(Pre-FEV_1_ × 100%)

and defined a change of 12% or greater as evidence of bronchodilator reversibility (BDR).

A fraction of exhaled nitric oxide (Feno) value of greater than 35 ppb was used to define the likely presence of eosinophilic airway inflammation.^19^ Additional details of spirometric methods are provided in the Methods section in this article's Online Repository.

### Early wheezing phenotypes at 6 to 81 months of age

Phenotypes of early childhood wheezing based on children's history of wheezing from 6 months to 81 months (7 years) of age were previously identified and published^2^: (1) *never/infrequent wheeze* (68% of children) was defined as an approximately 10% prevalence of wheezing at 6 months and decreasing prevalence of sporadic wheeze thereafter, including children who never reported wheeze; (2) *transient early wheeze* (10%) was defined as a 50% to 60% prevalence of wheeze up to 18 months, decreasing to low prevalence from 42 months; (3) *prolonged early wheeze* (8%) was defined as a peak prevalence of wheeze of around 65% at 30 months, decreasing to low prevalence from 69 months; (4) *intermediate-onset wheeze* (2%) was defined as a low prevalence of wheeze up to 18 months, increasing rapidly to high prevalence from age 42 months; (5) *late-onset wheeze* (5%) was defined as an approximately 20% prevalence of wheeze up to 42 months, increasing to more than 50% prevalence thereafter; and (6) *persistent wheeze* (7%) was defined as a 65% prevalence of wheeze at 6 months and approximately 90% prevalence thereafter.

### Latent class analysis

**Fig E1:**
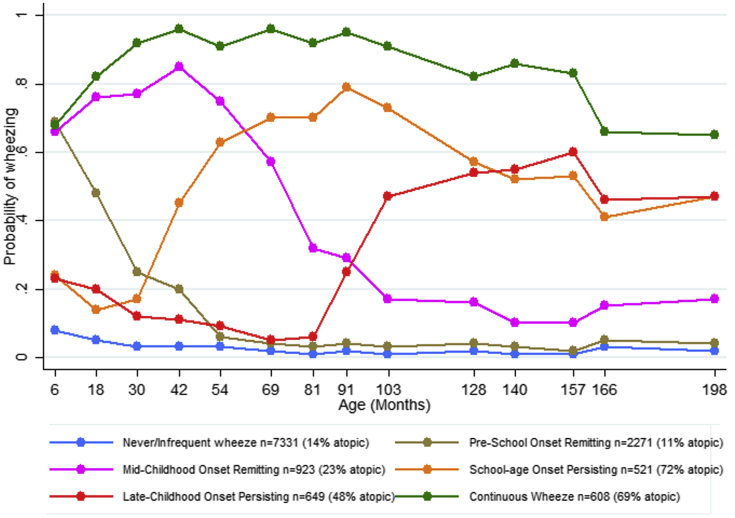
Estimated prevalence of wheezing at each time point from birth to 16½ years for each of the 6 wheezing phenotypes identified by using latent class analysis in 12,303 children with at least 2 observations of wheeze, including atopic status as a covariate in the model.

**Fig 1:**
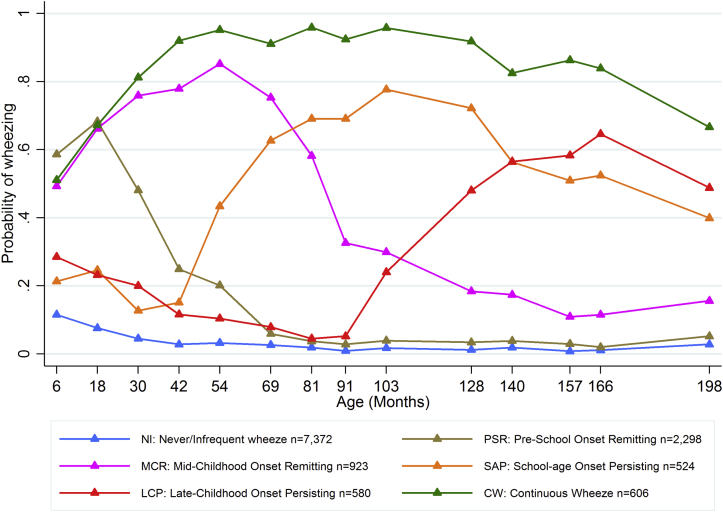
Estimated prevalence of wheezing at each time point from birth to 16½ years for each of the 6 wheezing phenotypes identified by using latent class analysis in 12,303 participants with at least 2 observations of wheeze.

**Fig 2:**
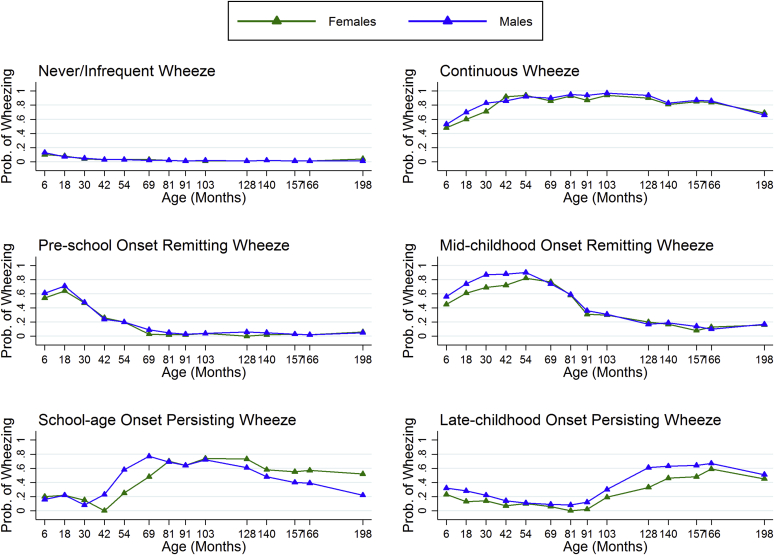
Estimated prevalence of wheezing at each time point from birth to 16½ years for each of the 6 wheezing phenotypes identified by using latent class analysis in 12,303 participants with at least 2 observations of wheeze by sex.

Parental reports of children's wheezing were obtained from questionnaires sent to mothers at approximately annual intervals from 6 to 198 months (16½ years) and used in a longitudinal latent class analysis to define phenotypes of wheezing, as previously described.^2^, ^20^ Starting with a model assuming 3 phenotypes, we compared models with increasing numbers of phenotypes using the Bayesian information criterion, the model entropy, and the Lo-Mendell-Rubin adjusted likelihood ratio tests to compare models with increasing numbers of phenotypes.^21^ The current article uses results based on analysis of participants with at least 2 responses to questionnaires about wheezing (n = 12,303 participants, excluding triples and quadruples) because these analyses yielded similar results to those based on complete cases (n = 3170 participants, see Table E1 in this article's Online Repository at www.jacionline.org). Based on the optimal 6-class model (Fig 1), we ran 2 additional models:

1. A sex-stratified model allowing the trajectory of wheezing phenotypes to differ between male and female subjects (Fig 2): We estimated the prevalence of wheezing in the extended wheezing phenotypes separately for male and female subjects and constructed single omnibus Wald tests (model test in Mplus) to test equality for each pair of wheezing phenotypes in turn (sex invariance of the optimal 6-class model). The null hypothesis of this test is no difference in the trajectory of wheezing for male and female subjects. We reported in Table I the mean difference of the probability of wheezing (based on the 14 time points) and the Wald test *P* value for each wheezing phenotype.

2. Model with atopy (skin prick testing at 7½ years) as a covariate (see Fig E1 in this article's Online Repository at www.jacionline.org): This model allows the shape of the wheezing phenotypes to differ from the model without atopy; we report prevalence of atopy by phenotype.

*P* values (χ^2^ test) from the logistic regression models using female subjects as the baseline group were reported in Table II to examine sex differences among characteristics of interest. Associations of potential confounders with wheezing phenotypes were examined by using multinomial logistic regression models weighted for the probability of each subject belonging to each phenotype (details are shown in the Methods section and Table E2 in this article's Online Repository at www.jacionline.org). We identified the following variables as being associated with at least 1 of the wheezing phenotypes and adjusted for them in subsequent analyses: sex, maternal lower education level, having at least 1 sibling (parity), maternal history of asthma or allergy, maternal smoking during pregnancy, maternal anxiety during pregnancy, low birth weight (<2.5 kg), preterm delivery (<37 weeks' gestation), and day care attendance during the first year.

All analyses were done with Mplus 7.1 software (2006; Muthén & Muthén, Los Angeles, Calif) and Stata 13.1 software (StataCorp, College Station, Tex).

## Results

### Characteristics of the study population

**Fig E2:**
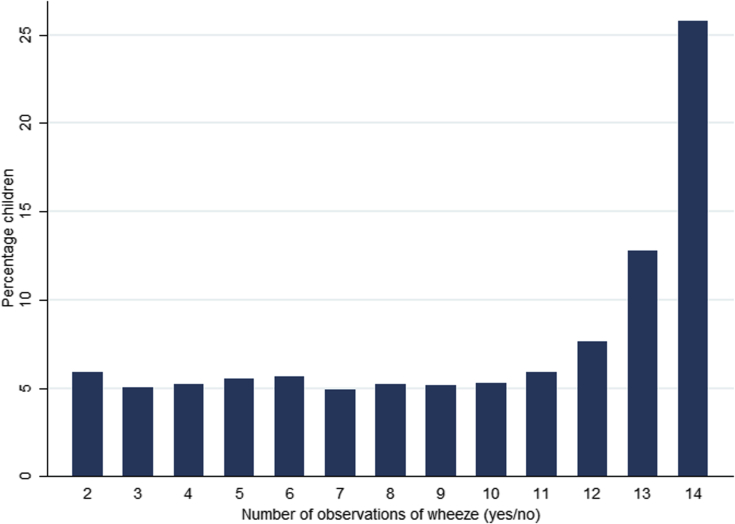
Distribution of 12,303 children with 2 to 14 (complete data) observations of wheeze.

Among 12,303 participants with at least 2 observations of wheezing from birth to 16½ years of age (the study population), 3,170 had complete data from all 14 questionnaires. Characteristics of participants with and without complete data are compared in Table E1. Participants with complete data were less likely to come from socially deprived backgrounds (lower prevalence of lower maternal education and maternal smoking) and had lower prevalence of reported wheezing in early childhood than participants with missing data. Corresponding results based on analysis of participants with complete reports of wheezing (n = 3,170) are available from the authors on request. Fig E2 in this article's Online Repository at www.jacionline.org shows the distribution of the number of observations of wheeze.

Table II reports characteristics of the study population by sex. A total of 6330 participants were male, and 5973 were female. A similar proportion of male and female subjects had an asthmatic or allergic mother (46.4% male subjects vs 46.0% female subjects), at least 1 sibling (55.4% vs 54.4%), low birth weight (4.9% vs 5.1%), and attended day care during the first year (6.1% vs 6.0%). Male subjects were more exposed to maternal smoking during pregnancy (27.2% vs 25.0%, *P* = .009), and more male subjects were born preterm (6.5% vs 5.1%, *P* = .002) compared with female subjects. The prevalence of wheezing was higher in male compared with female subjects from 6 months (29.7% vs 22.3%, *P* < .001) to 166 months (12.3% vs 9.7%, *P* < .001); however, the prevalence of wheezing was similar at 198 months (10.4% vs 10.6%, *P* = .88). The prevalence of atopy determined by using skin prick tests at 7½ years was higher in male than female subjects (24.5% vs 17.4%, *P* < .001).

### Late-childhood asthma outcomes

Table III shows the distributions of longitudinal and late-childhood asthma outcomes in the study population and restricted to participants with complete data on confounding variables. Among the phenotypes of wheezing from 0 to 7 years defined in our previous work,^2^ the most frequently occurring were transient early (1190 [10.2%] participants), prolonged early (822 [7.0%] participants), and persistent (899 [7.7%] participants) wheezing. Based on reports of doctor-diagnosed asthma ever by 7½ and 14 years, 1637 (23.2%) participants had experienced asthma by age 14 years, 707 (11.9%) had asthma by 7½ years but no asthma at 14 years (remittent asthma), 378 (6.3%) had incident asthma between 7½ and 14 years, and 446 (7.5%) had persistent asthma by 14 years. A total of 319 (8.3%) participants had BDR, and 680 (31.5%) participants had Feno values of greater than 35% ppb at 15 years. These proportions were similar when data was restricted to participants with complete data on confounders.

### Latent class analysis: Extended wheezing phenotypes at 0 to 16½ years of age

The best-fitting model based on 12,303 participants with reports of wheezing from 0 to 16½ years of age resulted in 6 extended wheezing phenotypes. Trajectories of prevalence of wheeze for each of these phenotypes are presented in Fig 1. We labeled the wheezing phenotypes as follows:

1. *never/infrequent wheeze* (estimated number of participants, 7372 [59.9%]) with approximately 10% prevalence of wheezing at 6 months and decreasing prevalence of sporadic wheeze thereafter, including participants who never reported wheeze;
2. *preschool-onset remitting wheeze* (2298 [18.7%]) with 50% to 60% prevalence of wheeze up to 18 months, decreasing to less than 10% prevalence from 69 months;
3. *midchildhood-onset remitting wheeze* (923 [7.5%]) with peak prevalence of wheeze around 84% at 54 months, decreasing to less than 20% from 128 months;
4. *school age–onset persisting wheeze* (524 [4.3%]) with less than 20% prevalence of wheeze up to 42 months, increasing rapidly to a peak prevalence of 87% at 103 months and then decreasing to 46% at 198 months;
5. *late childhood–onset persisting wheeze* (580 [4.7%]) with 32% prevalence at 6 months, decreasing up to 91 months and increasing rapidly to a peak prevalence of 71% at 166 months; and
6. *continuous wheeze* (606 [4.9%]) with approximately 41% prevalence of wheeze at 6 months and prevalence between 62% and 97% thereafter.

### Sex-specific wheezing phenotypes

Fig 2 shows trajectories of prevalence of wheeze estimated separately in male and female subjects for each phenotype. The sex-stratified wheezing phenotypes were similar to the extended phenotypes and were given the same labels (see above). The prevalence of the preschool-onset remitting phenotype was lower for female (17%) than male (21%) subjects (Table I). There were modest differences in prevalence of the school age–onset persisting phenotype (3% of female and 5% of male subjects). The prevalence of both the late childhood–onset persisting and continuous wheeze phenotypes was approximately 4% for female and 6% for male subjects. The never/infrequent wheeze phenotype had a prevalence of 64% in female subjects and 55% in male subjects.

The results from the Wald test strongly supported sex stratification of the school age–onset persisting (mean probability difference, 0.13; *P* = .0061) and late childhood–onset persisting (mean probability difference, 0.10; *P* < .0001) phenotypes (Table I), but no differences were observed for the other phenotypes, excluding never/infrequent wheeze. There was clear evidence (*P* = .004) that the never/infrequent wheeze phenotype differed between male and female subjects, but this difference was explained by between-sex differences in the prevalence of wheeze at the first (0.04) and last (0.03) time points within this phenotype. For other time points the between-sex differences were, on average, 0.01 or less, and the test *P* value increased to .38 in a sensitivity analysis excluding the first and last time points.

### Model including atopy

Fig E1 depicts the slightly modified wheezing phenotype model when including atopy as an additional covariate. The phenotypes with the highest prevalence of atopy were the school age–onset persisting (72% atopic) and continuous wheezing (69% atopic) phenotypes, followed by the late childhood–onset persisting (48% atopic) and midchildhood-onset remitting (23% atopic) phenotypes. The never/infrequent wheeze (14% atopic) and preschool-onset remitting (11% atopic) phenotypes had the lowest prevalence of atopic participants.

### Validation of the extended wheezing phenotypes

**Fig 3:**
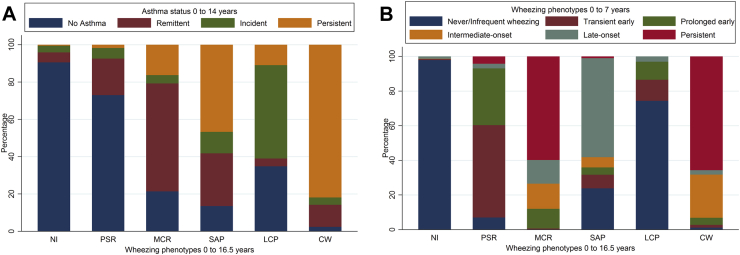
Validation of wheezing phenotypes at 0 to 16½ years with asthma status at 0 to 14 years **(A)** and wheezing phenotypes at 0 to 7 years **(B)** in 12,303 participants with at least 2 observations of wheeze. *CW*, Continuous wheeze; *LCP*, late childhood–onset persisting wheeze; *MCR*, midchildhood-onset remitting wheeze; *NI*, never/infrequent wheeze; *PSR*, preschool-onset remitting wheeze; *SAP*, school age–onset persisting wheeze.

To validate the extended wheezing phenotypes, we examined the proportion of participants classified as having no asthma, remittent asthma, incident asthma, and persistent asthma in each wheezing phenotype (Fig 3, *A*). As expected, the never/infrequent wheeze phenotype had the highest proportion (91%) of participants with no asthma, whereas the continuous wheeze phenotype had the highest proportion (82%) of participants with persistent asthma. The preschool-onset remitting phenotype mostly overlapped with no asthma (73%) and remittent asthma (20%), whereas the midchildhood-onset remitting phenotype mostly overlapped with remittent asthma (58%), no asthma (21%), and persistent asthma (16%). The school age–onset persisting phenotype mostly overlapped with persistent asthma (47%), remittent asthma (28%), and no asthma (14%). The late childhood−onset persisting phenotype mostly overlapped with incident asthma (50%), no asthma (35%), and persistent asthma (11%).

Fig 3, *B*, shows the proportion of participants classified with each of the previously published early childhood wheezing phenotypes for each extended wheezing phenotype. The never/infrequent wheeze phenotype had the highest proportion of early childhood never/infrequent wheezing (98% overlap), and the continuous wheeze phenotype overlapped mainly with early childhood persistent wheeze (66%) and intermediate-onset wheeze (25%). The preschool-onset remitting phenotype overlapped with early childhood transient early wheezing (53%) and prolonged early wheezing (33%). The midchildhood-onset remitting phenotype overlapped with early childhood persistent wheezing (60%) and with prolonged early, intermediate-onset, and late-onset wheezing (prevalence, 11% to 15%). The school age−onset persisting phenotype overlapped with early childhood late-onset wheezing (57%) and never/infrequent wheezing (24%). The late-childhood persisting phenotype mainly overlapped with early childhood never/infrequent wheezing (74%).

### Associations of extended wheezing phenotypes with doctor-diagnosed asthma, Feno, and BDR at 14 to 15 years of age

All wheezing phenotypes were associated with substantially higher odds of doctor-diagnosed asthma ever at 14 years compared with the never/infrequent wheeze phenotype (Table IV). Associations were particularly strong for continuous (adjusted odds ratio [OR], 368; 95% CI, 181-747), school age−onset persisting (OR, 50.0; 95% CI, 35.2-71.0), and midchildhood-onset remitting (OR, 25.1; 95% CI, 19.4-32.4) wheeze. The school age−onset persisting, late childhood−onset persisting, and continuous wheeze phenotypes were strongly associated with Feno values of 35 ppb or greater and BDR compared with never/infrequent wheeze. The strongest associations were for continuous wheeze with Feno values of 35 ppb or greater (adjusted OR, 6.74; 95% CI, 3.93-11.54) and school age−onset persisting wheeze with BDR (OR, 3.34; 95% CI, 2.08-5.35). Midchildhood-onset remitting wheeze was associated with Feno values of 35 ppb or greater (OR, 1.87; 95% CI, 1.23-2.84) and BDR (OR, 1.77; 95% CI, 1.11-2.82) compared with never/infrequent wheeze. There was little evidence for associations of preschool-onset remitting wheeze with either Feno values of 35 ppb or greater or BDR.

### Associations of extended wheezing phenotypes with lung function measures at 15 years

Continuous wheeze was associated with decrements of FEV_1_/FVC ratio (mean difference, −0.27 SDU; 95% CI, −0.45 to −0.09 SDU) and FEF_25-75_ (mean difference, −0.33 SDU; 95% CI, −0.51 to −0.15 SDU) compared with never/infrequent wheeze (Table V). The preschool- and midchildhood-onset remitting phenotypes were associated with a small decrement of FEV_1_/FVC ratio (mean difference, −0.15 SDU; [95% CI, −0.25 to −0.05 SDU] and −0.22 SDU [95% CI, −0.36 to −0.08 SDU]) and FEF_25-75_ (mean difference, −0.14 SDU [95% CI, −0.24 to −0.04 SDU] and −0.16 SDU [95% CI, −0.30 to −0.01 SDU], respectively) compared with never/infrequent wheeze. Midchildhood-onset remitting was also associated with an increment of FVC (0.20 SDU; 95% CI, 0.06-0.34 SDU) compared with never/infrequent wheeze. School-age–onset persisting wheeze was associated with small decrements of FEV_1_/FVC ratio (−0.22 SDU; 95% CI, −0.39 to −0.04 SDU), and late childhood–onset persisting wheeze was associated with small decrements of FEF_25-75_ (−0.21 SDU; 95% CI, −0.38 to −0.04 SDU) compared with never/infrequent wheeze.

Crude associations are presented in Tables E3 and E4 in this article's Online Repository at www.jacionline.org: the estimates were little attenuated by adjustment for potential confounders. Corresponding association with lung function measures at 8½ years are presented in Table E5 in this article's Online Repository at www.jacionline.org. The association of midchildhood-onset remitting wheeze with FVC at 15 years was not observed at 8 years. However, associations of preschool-onset remitting, midchildhood-onset remitting, school age–onset persisting, and continuous wheeze with FEV_1_/FVC ratio were observed at 8 years and appeared attenuated at 15 years, whereas associations of preschool-onset remitting, midchildhood–onset remitting, late childhood–onset persisting, and continuous wheeze with FEF_25-75_ were observed at 8 years and increased at 15 years. Table E6 in this article's Online Repository at www.jacionline.org reports the associations with presalbutamol lung function measures at 15 years, and Table E7 in this article's Online Repository at www.jacionline.org reports the associations with presalbutamol lung function measures at 15 years further adjusted for lung function measures at 8½ years.

## Discussion

### Main findings

Based on analyses of wheezing measured on 14 occasions between the ages 6 and 198 months in 12,303 participants in the ALSPAC birth cohort study, we identified 6 phenotypes of childhood wheezing and quantified their associations with doctor-diagnosed asthma and objective measures of Feno, BDR, and lung function in late childhood. Almost 60% of participants were classified as having never or infrequently wheezed, with wheezing in these participants being sporadic and usually early. Two of the wheezing phenotypes identified were characterized by remission of symptoms in later childhood, and 3 were associated with persistent symptoms from onset in midchildhood to late childhood or continuous symptoms from infancy. Compared with the never/infrequent wheezing phenotype, those phenotypes with remitting symptoms (preschool-onset remitting wheeze and midchildhood-onset remitting wheeze), comprising 27% of the study population, were associated with lung function deficits in adolescence, with the later-onset group also having evidence of eosinophilic inflammation and BDR at age 15 years. All 3 phenotypes with symptoms persisting to adolescence were characterized by strong associations with diagnosed asthma, BDR, and high Feno measurements, with continuous wheeze being the most strongly associated with these outcomes. These associations are summarized in Table VI.

### Results in the context of the existing literature

Our approach to classifying childhood wheezing up to age 7 years by its temporal patterns using latent class analysis^2^ has been replicated^15^ and validated^4^, ^5^ by independent studies and has utility in identifying clinically relevant phenotypes with high sensitivity and specificity.^9^ However, there is uncertainty about whether phenotypes classified this way represent distinct pathophysiologic processes leading to different long-term outcomes or whether outcome variation is related to the duration and severity of a single disease process.

Several cohort studies of asthma beginning in childhood have followed lung function development through to adult life. In the Melbourne Asthma Study^22^ there was a relationship between the severity of wheezing illness in early childhood and the probability of persistent asthma in adulthood and obstructive airway function to age 50 years. In keeping with longitudinal lung function measurements from midchildhood (age 9 years) in the Dunedin cohort^1^ and from infancy in the Tucson Children's Respiratory Study,^23^ it appeared that lung function deficits were already established by school age and adult obstructive airway function was explained by tracking of these deficits into adult life rather than by a more rapid decrease of adult lung function. Studies with lung function measurements from early infancy have reported associations of lung function deficits shortly after birth with the development of asthma in childhood,^24^, ^25^ but there is inconsistency regarding the trajectories of lung function development from birth to school age between these and the Tucson study, which suggested that children who had wheezing and asthma had near-normal lung function at birth but airway obstruction by 6 years, which then tracked to adulthood. In the Danish COPSAC study low lung function was present soon after birth, with further deviation from lung function development in healthy infants occurring during the first 7 years. The authors estimated that 40% of the lung function deficit at 7 years in children with asthma was explained by low lung function at birth and the remaining 60% by early-life developmental processes. They also reported that lung function–associated genetic polymorphisms in adults were associated with lung function development form birth to 7 years but not with neonatal lung function,^26^ suggesting that the mechanisms influencing these 2 aspects are different.

Our data are consistent with the notion that early onset of wheezing symptoms is associated with establishment of low lung function by school age, which persists to adolescence, whereas late-onset wheezing (after 7 years) was not associated with postbronchodilator airway obstruction by age 15 years. All phenotypes with evidence of wheezing during the first 7 years were associated with airway obstruction (low FEV_1_/FVC ratio) in adolescence in contrast to the late childhood–onset persisting phenotype. Although the greatest deficit was present in the group with early wheeze onset (6 months after birth) that persisted until adolescence, this supports onset of wheezing illness in early childhood having a greater influence than its duration after onset and hence a window of development when intervention might be possible.

Our findings are compatible with a multifactorial etiology of low lung function in adolescence associated with wheezing phenotypes and asthma in childhood. It is likely that airway developmental abnormalities around the time of birth contribute both to the likelihood of asthma and its consequences. In the Tucson study those with the lowest airway function soon after birth had transient wheezing, and their lung function appeared to regress to the mean during the first 6 years,^27^ although the lowest quartile of lung function at birth was associated with low lung function at age 22 years in the same cohort.^23^ Transient wheeze in the Melbourne study and remitting wheeze in the Dunedin study were both associated with smaller deficits of lung function in adulthood than those with persistent asthma phenotypes.

What causes lung function to decrease during the preschool years in children with wheezing illnesses is a matter of speculation. Airway inflammation has been reported to be associated with signs of remodeling, even in early childhood,^28^ and it is conceivable that untreated airway inflammation in early life could result in progressive irreversible obstruction, although the processes in infancy probably differ from those associated with asthma in older children.^29^ It is notable that intervention with inhaled corticosteroids to treat mild-to-moderate asthma in the Childhood Asthma Management Program study after age 5 years^30^ and children at high risk for asthma aged 2 to 3 years in the Prevention of Early Asthma in Kids (PEAK) study^31^ did not provide evidence that anti-inflammatory treatment was effective in improving lung function in later childhood in either of these populations.

Some of the lung function deficits that we observed could be accounted for by current asthma at the time of measurement, with evidence of associated asthma, BDR, and eosinophilic airway inflammation present in all wheeze phenotypes, except preschool-onset remitting wheeze. However, there was still evidence of airway obstruction after bronchodilation in some of these subjects, particularly those with persisting wheeze, suggesting that at least a component of airway obstruction in these participants was irreversible. We cannot infer from these data the nature of the complex relationship between airway development, asthma, airway inflammatory responses, and remodeling in the causal pathway of low adult lung function. It appears that wheeze that starts early in life (in the preschool years) and persists to adolescence is most strongly associated with airway obstruction in adolescence, and a focus on identifying factors in this population that lead to persistence of symptoms might help to identify targets for intervention other than with corticosteroids.

We found a higher prevalence of atopy in the wheezing phenotypes that were more strongly associated with persistence of wheezing after onset (including continuous wheeze from infancy) than with remitting phenotypes. An association of atopy with persistent asthma symptoms has been reported previously^32^, ^33^ and might be explained by the association of eosinophilic airway inflammation with asthma severity and persistence in childhood.^34^, ^35^ Eosinophilic airway inflammation has been identified in preschool children with confirmed wheezing,^28^ and high Feno levels are associated with persistent compared with transient wheeze in this age group.^36^

Attention has recently focused on better characterization of the temporal pattern of allergic sensitization in children, and it is possible that different pathways of sensitization are associated with different pathophysiology of wheezing illnesses in early childhood. Belgrave and others have reported associations of a multiple early allergy phenotype^37^ with increased airways resistance through childhood to age 11 years.^38^ Joint latent class models of atopy and wheezing have also shown differences in associations of early-life exposures between wheezing phenotypes associated with high and low levels of atopy.^39^ We were limited in our ability to replicate these findings in our cohort because we had only 1 measurement of sensitization at age 7 years.

We found strong evidence of between-sex differences for the school age–onset and late childhood–onset persisting phenotypes. For the school age–onset persisting phenotype, with wheeze starting after 3½ years, the probability of wheezing between 9 and 16 years decreased substantially more in male subjects (0.72 to 0.22) than in female subjects (0.74 to 0.52). Thus this phenotype, with wheeze starting after 3½ years, might be considered to have a high probability of remission in male subjects during adolescence. Consistent with other studies, we observed an early male predominance of wheezing, but this had equalized by age 16 years. A sex switch toward female predominance of asthma in early adulthood has been reported in other studies,^1^, ^40^ and it has been suggested that hormonal influences might play a role in incident female asthma during and after puberty.^41^, ^42^ Most of the female participants in our study had reported menarche by the age of 16 years, but we did not see strong evidence for a large increase in asthma prevalence in female subjects by this age, perhaps suggesting that if hormonal effects are causal in promoting asthma in female subjects, they are unlikely to be expressed in the short term but might exert their influences through indirect mechanisms with a longer time lag.

### Strengths and limitations

An advantage of our study is the availability of data from a large, unselected, population-based cohort followed to late adolescence. For comparison, the Tucson Study reported on 826 children during the first 6 years,^3^ and the Dunedin study reported on 613 subjects with complete respiratory data at 9 to 26 years.^1^ The Perth study of infant lung function reported outcomes at 11 years in 183 infants,^43^ and a cohort of 2860 infants in Perth reported on asthma to age 6 years.^44^

Wheeze reported on up to 14 occasions (from 6 months to nearly 16 years) was used in a data-driven (latent-class) approach to derive childhood wheezing phenotypes, so that preconceptions about phenotype structure did not affect the derived phenotype groups. We were able to look at associations between the derived childhood wheezing phenotypes and different measures of lung function at 15 years, despite the fact that some phenotypes represented relatively small proportions of children.

There are some limitations to our study design and findings. The reports of wheezing used in the latent class analyses to derive wheezing phenotypes were not always contemporaneous with measurements of clinical outcomes: in particular, the latest measurements of doctor-diagnosed asthma and lung function were at ages 14 and 15 years, respectively. We think it is likely that associations of these outcomes with wheezing phenotypes will be similar to those had the outcomes been measured at the time of the last wheezing measurement at age 16½ years because wheezing phenotypes were derived from all available data from 6 months onward and therefore unlikely to be markedly influenced by wheezing at a single time point.

A major limitation of latent class models is their application to the clinical setting because children with wheezing cannot be assigned prospectively to a particular phenotype, which has been defined by *post hoc* data analysis. However, by demonstrating the existence of different trajectories of wheezing illnesses through childhood and their associated outcomes, we aim to identify those phenotypes associated with adverse long-term outcomes that might be amenable to intervention in early life. The challenge is then to find associated biomarkers of these disease types that can be applied to distinguish early wheeze that persists from that which remits, investigate the pathophysiologic pathways that exist at an early stage in these evolving phenotypes, and use this information to derive interventions that might be phenotype specific. Finally, we have been unable to verify the findings of our study in an independent cohort, and replication will be an essential first step to realizing the utility of hypothesis generation based on our phenotypes.

### Conclusion

This study adds to our previous description of wheezing phenotypes in children by extending knowledge of variation in the natural history of wheeze from infancy through the critical adolescent transition period to adulthood, during which the sex distribution of asthma changes and lung development nears its peak. We confirmed the importance of early childhood wheezing illness in lung function development and identified the temporal wheezing patterns most strongly associated with airway obstruction as wheeze starting before the age of 4 years and persisting throughout childhood. The challenge is to translate this knowledge by identifying clinically useful biomarkers associated with disease phenotypes that can be measured in early childhood and hence to enable the study of disease pathways associated with unfavorable prognosis and development of targeted interventions to modify the natural history of wheezing illnesses in young children, a population in whom treatment with corticosteroids appears to be ineffective in disease modification.

## Figures and Tables

**Table E1:** Characteristics of children with at least 2 observations of wheeze (study population, n = 12,303) who did and did not have complete data on wheezing

	Children with complete data on wheezing (n_1_ = 3,170)		Children with 2-13 observations of wheezing (n_2_ = 9,133)		Two-sample test of proportions
	No./total	Percent	No./total	Percent	*P* value
Potential confounders					
Male sex	1,560/3,170	49.2	4,770/9,133	52.2	.003
Lower maternal education∗	1,547/3,151	49.1	5,614/8,184	68.6	<.0001
Having ≥1 sibling (parity)	1,597/3,118	51.2	4,551/8,072	56.4	<.0001
Maternal history of asthma or allergy	1,510/3,107	48.6	3,629/8,025	45.2	.001
Maternal smoking during pregnancy	439/3,127	14.0	2,495/8,096	30.8	<.0001
Maternal anxiety during pregnancy†	479/3,020	15.9	1,831/7,520	24.3	<.0001
Low birth weight (<2.5 kg)	134/3,134	4.3	441/8,397	5.3	.032
Preterm delivery (<37 wk)	153/3,170	4.8	529/8,504	6.2	.004
Maternal smoking during first year	411/3,122	13.2	2,118/7,690	27.5	<.0001
Day care attendance during first year	242/3,124	7.7	399/7,505	5.3	<.0001
Variables used in the latent class model					
Reported wheezing at:					
6 mo	709/3,170	22.4	2,165/7,834	27.6	<.0001
18 mo	780/3,170	24.6	2,205/7,691	28.7	<.0001
30 mo	612/3,170	19.3	1,632/6,775	24.1	<.0001
42 mo (3½ y)	487/3,170	15.4	1,269/6,803	18.7	<.0001
54 mo	505/3,170	15.9	1,269/6,221	20.4	<.0001
69 mo	435/3,170	13.7	893/5,426	16.5	.0007
81 mo	393/3,170	12.4	734/5,225	14.0	.70
91 mo	334/3,170	10.5	544/5,032	10.8	.14
103 mo (8½ y)	386/3,170	12.2	664/4,990	13.3	.41
128 mo	376/3,170	11.9	559/4,475	12.5	.37
140 mo	360/3,170	11.4	509/4,229	12.0	.83
157 mo	338/3,170	10.7	402/3,828	10.5	.44
166 mo	339/3,170	10.7	424/3,760	11.3	.86
198 mo (16½ y)	335/3,170	10.6	257/2,465	10.4	.39
Atopy at 7½ y (skin prick test)	524/2,433	21.5	879/4,258	20.6	.70

∗ Educated to General Certificate of Education level (school-leaving certificate) or lower.

† Defined as being in the fourth quantile of the Crown-Crisp Experiential Index.

**Table E2:** Associations of possible confounders with wheezing phenotypes at 0 to 16½ years and asthma at 14 years (based on 12,303 children with at least 2 observations of wheezing)

	Adjusted multinomial OR (95% CI)						Adjusted OR (95% CI) for ever doctor-diagnosed asthma at 14 y (n = 5,838)
	Preschool-onset remitting wheeze∗ (n = 1,605)	Midchildhood-onset remitting wheeze∗ (n = 620)	School-age–onset persisting wheeze∗ (n = 346)	Late childhood–onset persisting wheeze∗ (n = 324)	Continuous wheeze∗ (n = 420)	Heterogeneity *P* value†	
Demographic, maternal, pregnancy, and child characteristics (adjusted by each other)							
Male sex	1.39 (1.25-1.55)	1.41 (1.21-1.66)	1.54 (1.26-1.89)	1.32 (1.09-1.60)	1.80 (1.48-2.19)	.13	1.26 (1.11-1.42)
Maternal lower education level‡	0.96 (0.86-1.08)	1.22 (1.02-1.45)	0.92 (0.74-1.13)	0.94 (0.77-1.14)	0.95 (0.77-1.16)	.11	1.13 (1.00-1.28)
Parity	1.24 (1.11-1.38)	1.38 (1.17-1.62)	0.88 (0.72-1.07)	1.02 (0.85-1.24)	1.37 (1.12-1.67)	1.2E-03	0.95 (0.84-1.07)
Maternal history of asthma or allergy	1.27 (1.14-1.42)	1.65 (1.40-1.93)	1.47 (1.20-1.80)	1.42 (1.17-1.72)	2.30 (1.88-2.81)	1.9E-06	1.53 (1.35-1.73)
Maternal smoking during pregnancy	1.33 (1.17-1.51)	1.46 (1.22-1.75)	1.23 (0.97-1.56)	1.24 (0.99-1.56)	1.38 (1.10-1.72)	.71	1.19 (1.01-1.39)
Maternal anxiety during pregnancy	1.20 (1.14-1.26)	1.25 (1.16-1.35)	1.12 (1.02-1.23)	1.14 (1.04-1.24)	1.29 (1.18-1.41)	.08	1.13 (1.07-1.20)
Perinatal characteristics adjusted by demographic, maternal, pregnancy, and child characteristics							
Low birth weight (<2.5 kg)§	1.00 (0.73-1.37)	1.14 (0.73-1.78)	1.47 (0.86-2.52)	1.56 (0.93-2.60)	1.63 (1.00-2.64)	.26	1.11 (0.77-1.61)
Preterm delivery (<37 wk)§	1.35 (1.07-1.70)	1.43 (1.03-2.00)	1.04 (0.65-1.67)	0.94 (0.59-1.50)	1.63 (1.11-2.39)	.30	1.16 (0.87-1.56)
Postnatal characteristics adjusted by demographic, maternal, pregnancy, child, perinatal, and other postnatal characteristics							
Maternal smoking during first year	1.22 (0.98-1.51)	1.15 (0.84-1.58)	0.96 (0.64-1.44)	1.01 (0.69-1.49)	1.09 (0.74-1.61)	.80	1.06 (0.82-1.37)
Day care attendance during first year	1.28 (1.02-1.60)	0.97 (0.66-1.41)	0.72 (0.44-1.18)	0.92 (0.59-1.42)	1.07 (0.70-1.63)	.16	0.90 (0.70-1.17)

∗ Compared with never/infrequent wheezing (n = 6,305) and using each child's phenotype probability as weights.

† χ^2^ Test across phenotypes.

‡ Educated to school-leaving certificate at 16 years (General Certificate of Education level) or lower.

§ Low birth weight was also adjusted by preterm delivery, but preterm delivery was not adjusted for low birth weight (because birth weight does not influence gestational age).

**Table E3:** Crude associations of wheezing phenotypes at 0 to 16½ years with doctor-diagnosed asthma ever, Feno values of 35 ppb or greater, and BDR of greater than 12% at 14 to 15 years (based on 12,303 children with ≥2 observations of wheeze)

Wheezing phenotype at 0-16½ y	Doctor-diagnosed asthma ever at 14 y		Feno ≥35 ppb at 14-15 y		BDR >12% at 15 y	
	No. of asthmatic patients/total∗	OR (95% CI)	n_1_/total∗	OR (95% CI)	n_2_/total∗	OR (95% CI)
Never/infrequent wheeze	272/3,661	1 (reference)	290/1,157	1 (reference)	139/2,003	1 (reference)
Preschool-onset remitting wheeze	173/870	2.74 (2.23-3.36)	61/243	1.06 (0.79-1.44)	33/472	1.10 (0.76-1.59)
Midchildhood-onset remitting wheeze	231/337	25.0 (19.4-32.2)	42/102	1.93 (1.28-2.90)	21/204	1.66 (1.04-2.63)
School-age–onset persisting wheeze	187/223	49.9 (35.3-70.7)	57/82	6.39 (3.95-10.34)	27/127	3.28 (2.06-5.22)
Late childhood–onset persisting wheeze	161/250	19.8 (15.0-26.1)	38/66	3.67 (2.30-5.85)	19/131	2.10 (1.26-3.48)
Continuous wheeze	246/254	363 (179-735)	49/71	6.95 (4.13-11.72)	19/126	2.36 (1.40-3.98)

*n*_*1*_, Number of children with Feno values of 20 ppb or greater; *n*_*2*_, number of children with BDR of greater than 12%.

∗ Number of children with data available (approximated from modal assignment).

**Table E4:** Crude association of wheezing phenotypes at 0 to 16½ years with lung function measures at 15 years (*z* scores) based on 12,303 children with at least 2 observations of wheezing

Wheezing phenotype at 0-16½ y	FEV_1_ (SDU) at 15 y		FVC (SDU) at 15 y		FEV_1_/FVC ratio (SDU) at 15 y		FEF_25-75_ (SDU) at 15 y	
	No.∗	Mean difference (95% CI)	No.∗	Mean difference (95% CI)	No.∗	Mean difference (95% CI)	No.∗	Mean difference (95% CI)
Never/infrequent wheeze	1,997	0 (reference)	2,094	0 (reference)	1,997	0 (reference)	2,094	0 (reference)
Preschool-onset remitting	463	−0.02 (−0.12 to 0.07)	477	0.05 (−0.04 to 0.14)	463	−0.15 (−0.24 to −0.05)	477	−0.13 (−0.22 to −0.03)
Midchildhood-onset remitting wheeze	203	0.09 (−0.05 to 0.23)	209	0.21 (0.07 to 0.35)	203	−0.23 (−0.37 to −0.08)	209	−0.14 (−0.28 to −0.00)
School-age–onset persisting wheeze	127	0.08 (−0.10 to 0.26)	131	0.18 (0.00 to 0.35)	127	−0.21 (−0.39 to −0.04)	131	−0.14 (−0.32 to 0.03)
Late childhood–onset persisting wheeze	130	−0.02 (−0.19 to 0.15)	135	0.02 (−0.14 to 0.19)	130	−0.08 (−0.25 to 0.08)	135	−0.21 (−0.37 to −0.04)
Continuous wheeze	126	−0.08 (−0.26 to 0.10)	131	0.04 (−0.14 to 0.22)	126	−0.26 (−0.44 to −0.08)	131	−0.30 (−0.48 to −0.12)

∗ Number of children with data available (approximated from modal assignment).

**Table E5:** Adjusted associations of wheezing phenotypes at 0 to 16½ years with lung function measures at 8 years (*z* scores) in participants with at least 2 observations of wheeze

Wheezing phenotype at 0-16½ years	FEV_1_ (SDU) at 8 y		FVC (SDU) at 8 y		FEV_1_/FVC ratio (SDU) at 8 y		FEF_25-75_ (SDU) at 8 y	
	No.∗	Adjusted mean difference (95% CI)†	No.∗	Adjusted mean difference (95% CI)†	No.∗	Adjusted mean difference (95% CI)†	No.∗	Adjusted mean difference (95% CI)†
Never/infrequent wheeze	3,394	0 (reference)	3,452	0 (reference)	3,295	0 (reference)	3,452	0 (reference)
Preschool-onset remitting wheeze	872	−0.20 (−0.28 to −0.13)	884	−0.06 (−0.13 to 0.02)	851	−0.24 (−0.31 to −0.17)	884	−0.28 (−0.35 to −0.21)
Midchildhood-onset remitting wheeze	336	−0.23 (−0.34 to −0.12)	341	−0.01 (−0.11 to 0.10)	329	−0.37 (−0.48 to −0.26)	341	−0.41 (−0.51 to −0.30)
School-age–onset persisting wheeze	219	−0.30 (−0.43 to −0.16)	222	−0.01 (−0.14 to 0.12)	215	−0.44 (−0.58 to −0.31)	222	−0.48 (−0.62 to −0.35)
Late childhood–onset persisting wheeze	212	−0.23 (−0.36 to −0.11)	215	−0.08 (−0.21 to 0.05)	207	−0.21 (−0.34 to −0.08)	215	−0.30 (−0.43 to −0.18)
Continuous wheeze	245	−0.40 (−0.53 to −0.27)	249	0.10 (−0.03 to 0.23)	236	−0.78 (−0.91 to −0.65)	249	−0.75 (−0.88 to −0.62)

∗ Number of participants with data available (approximated from modal assignment).

† Adjusted for sex, parity, maternal history of asthma or allergy, maternal smoking and anxiety during pregnancy, preterm delivery, low birth weight, and day care attendance during first year.

**Table E6:** Adjusted associations of wheezing phenotypes at 0 to 16½ years with presalbutamol lung function measures at 15 years (*z* scores) in participants with at least 2 observations of wheeze

Wheezing phenotype at 0-16½ y	FEV_1_ (SDU) at 15 y		FVC (SDU) at 15 y		FEV_1_/FVC ratio (SDU) at 15 y		FEF_25-75_ (SDU) at 15 y	
	No.∗	Adjusted mean difference (95% CI)†	No.∗	Adjusted mean difference (95% CI)†	No.∗	Adjusted mean difference (95% CI)†	No.∗	Adjusted mean difference (95% CI)†
Never/infrequent wheeze	2,064	0 (reference)	2,092	0 (reference)	2,064	0 (reference)	2,091	0 (reference)
Preschool-onset remitting wheeze	473	−0.06 (−0.16 to 0.03)	477	0.05 (−0.05 to 0.14)	473	−0.21 (−0.31 to −0.12)	476	−0.16 (−0.25 to −0.06)
Midchildhood-onset remitting wheeze	207	−0.03 (−0.17 to 0.11)	209	0.16 (0.02 to 0.30)	207	−0.35 (−0.49 to −0.22)	209	−0.25 (−0.39 to −0.11)
School-age–onset persisting wheeze	129	−0.16 (−0.33 to 0.02)	131	0.08 (−0.10 to 0.25)	129	−0.45 (−0.62 to −0.28)	131	−0.40 (−0.57 to −0.22)
Late childhood–onset persisting wheeze	135	−0.20 (−0.36 to −0.03)	135	−0.04 (−0.21 to 0.13)	135	−0.29 (−0.45 to −0.13)	135	−0.33 (−0.50 to −0.17)
Continuous wheeze	128	−0.23 (−0.41 to −0.04)	130	0.04 (−0.14 to 0.22)	128	−0.54 (−0.72 to −0.37)	130	−0.53 (−0.71 to −0.35)

∗ Number of participants with data available (approximated from modal assignment).

† Adjusted for sex, parity, maternal history of asthma or allergy, maternal smoking and anxiety during pregnancy, preterm delivery, low birth weight, and day care attendance during first year.

**Table E7:** Adjusted associations of wheezing phenotypes at 0 to 16½ years with lung function measures at 15 years (presalbutamol *z* scores) further adjusted by lung function measures at 8 years (presalbutamol *z* scores) in participants with at least 2 observations of wheeze

Wheezing phenotype at 0-16½ y	FEV_1_ (SDU) at 15 y		FVC (SDU) at 15 y		FEV_1_/FVC ratio (SDU) at 15 y		FEF_25-75_ (SDU) at 15 y	
	No.∗	Adjusted mean difference (95% CI)†	No.∗	Adjusted mean difference (95% CI)†	No.∗	Adjusted mean difference (95% CI)†	No.∗	Adjusted mean difference (95% CI)†
Never/infrequent wheeze	1,784	0 (reference)	1,826	0 (reference)	1,743	0 (reference)	1,825	0 (reference)
Preschool-onset remitting wheeze	415	0.02 (−0.08 to 0.11)	422	0.08 (−0.01 to 0.17)	407	−0.16 (−0.25 to −0.07)	421	−0.03 (−0.12 to 0.06)
Midchildhood-onset remitting wheeze	183	0.08 (−0.05 to 0.22)	187	0.17 (0.04 to 0.31)	179	−0.22 (−0.36 to −0.09)	187	−0.02 (−0.15 to 0.11)
School-age–onset persisting wheeze	110	−0.05 (−0.22 to 0.13)	113	0.11 (−0.06 to 0.28)	110	−0.33 (−0.50 to −0.16)	113	−0.19 (−0.36 to −0.03)
Late childhood–onset persisting wheeze	117	−0.07 (−0.24 to 0.09)	118	0.02 (−0.14 to 0.18)	117	−0.21 (−0.37 to −0.05)	118	−0.17 (−0.32 to −0.01)
Continuous wheeze	106	−0.04 (−0.22 to 0.14)	110	0.06 (−0.12 to 0.23)	103	−0.25 (−0.42 to −0.07)	110	−0.14 (−0.31 to 0.03)

∗ Number of participants with data available (approximated from modal assignment).

† Adjusted for sex, parity, maternal history of asthma or allergy, maternal smoking and anxiety during pregnancy, preterm delivery, low birth weight, and day care attendance during first year and additionally for lung function at 8 years.

**Table I:** Distribution of the extended phenotypes of wheezing from 0 to 16½ years among female (n = 5973) and male (n = 6330) members of the study population

Phenotype	Female subjects, no. (%)∗	Male subjects, no. (%)∗	Mean probability difference†	*P* value, Wald test
Never/infrequent wheeze	3800 (63.6)	3499 (55.3)	0.01	.004
Preschool-onset remitting wheeze	1020 (17.1)	1333 (21.1)	0.03	.33
Midchildhood-onset remitting wheeze	444 (7.4)	442 (7)	0.06	.53
School-age–onset persisting wheeze	190 (3.2)	312 (4.9)	0.13	.0061
Late childhood–onset persisting wheeze	260 (4.4)	357 (5.6)	0.10	<.0001
Continuous wheeze	259 (4.3)	387 (6.1)	0.04	.26

∗ Most probable phenotype based on model estimated probabilities, including sex as a known class.

† For each phenotype, the difference between male and female subjects of the probability of wheezing at each time point were calculated and then averaged.

**Table II:** Characteristics of participants with at least 2 observations of wheeze (study population, n = 12,303) by sex

	Participants with 2-14 observations of wheezing (n = 12,303)				
	Male subjects (n_1_ = 6,330)		Female subjects (n_2_ = 5,973)		*P* value (χ^2^ test)
	No./total	Percent	No./total	Percent	
Potential confounders					
Lower maternal education∗	3,715/5,848	63.5	3,446/5,487	62.8	.43
Having ≥1 sibling (parity)	3,202/5,777	55.4	2,946/5,413	54.4	.29
Maternal history of asthma or allergy	2,652/5,721	46.4	2,487/5,411	46.0	.68
Maternal smoking during pregnancy	1,570/5,774	27.2	1,364/5,449	25.0	.009
Maternal anxiety during pregnancy†	1,193/5,434	22.0	1,117/5,106	21.9	.92
Low birth weight (<2.5 kg)	293/5,951	4.9	282/5,580	5.1	.75
Preterm delivery (<37 wk)	391/6,023	6.5	291/5,651	5.1	.002
Maternal smoking during first year	1,334/5,557	24.0	1,195/5,255	22.7	.12
Day care attendance during first year	333/5,493	6.1	308/5,136	6.0	.89
Variables used in latent class model					
Reported wheezing at:					
6 mo	1,683/5,673	29.7	1,191/5,331	22.3	<.001
18 mo	1,730/5,622	30.8	1,255/5,239	24.0	<.001
30 mo	1,312/5,144	25.5	932/4,801	19.4	<.001
42 mo (3½ y)	1,000/5,159	19.4	756/4,814	15.7	<.001
54 mo	1,035/4,861	21.3	739/4,530	16.3	<.001
69 mo	767/4,434	17.3	561/4,162	13.5	<.001
81 mo	646/4,321	15.0	481/4,074	11.8	<.001
91 mo	526/4,218	12.5	352/3,984	8.8	<.001
103 mo (8½ y)	626/4,160	15.0	424/4,000	10.6	<.001
128 mo	567/3,848	14.7	368/3,797	9.7	<.001
140 mo	508/3,692	13.8	361/3,707	9.7	<.001
157 mo	431/3,490	12.3	309/3,508	8.8	<.001
166 mo	427/3,475	12.3	336/3,455	9.7	<.001
198 mo (16½ y)	286/2,739	10.4	306/2,896	10.6	.88
Atopy at 7½ y (skin prick test)	825/3,364	24.5	578/3,327	17.4	<.001

∗ Educated to the General Certificate of Education level (school-leaving certificate) or lower.

† Anxious mothers were defined as being in the fourth quantile of the Crown-Crisp Experiential Index.

**Table III:** Longitudinal and late-childhood outcomes in the study population and restricted to participants with complete data on confounders

	All participants in study population (n = 12,303)		Restricted to participants with complete data on confounders (n = 8,925)§	
	Total no.∗	No. (%) or IQR	Total no.∗	No. (%) or IQR
Longitudinal outcomes				
Wheezing phenotypes at 0-7 y	11,674		8,925	
Never/infrequent wheezing		7,961 (68.2)		6,154 (69.0)
Transient early wheezing		1,190 (10.2)		884 (9.9)
Prolonged early wheezing		822 (7.0)		623 (7.0)
Intermediate-onset wheezing		275 (2.4)		223 (2.5)
Late-onset wheezing		527 (4.5)		410 (4.6)
Persistent wheezing		899 (7.7)		631 (7.1)
Asthma status at 0-14 y	5,960		5,053	
No asthma		4,429 (74.3)		3,774 (74.7)
Asthma by 7½ y but no asthma at 14 y		707 (11.9)		581 (11.5)
Incident asthma between 7½ and 14 y		378 (6.3)		317 (6.3)
Persistent asthma by 14 y		446 (7.5)		381 (7.5)
Late-childhood outcomes				
Ever doctor-diagnosed asthma by 14 y	7,052	1,637 (23.2)	5,595	1,270 (22.7)
BDR at 15 y‡	3,831	319 (8.3)	3,063	258 (8.4)
Feno ≥35% at 14-15 y	2,159	680 (31.5)	1,721	537 (31.2)
*z* Score FEV_1_ (SDU) at 15 y†	3,657	−0.61 to 0.64	3,046	−0.62 to 0.64
*z* Score FVC (SDU) at 15 y†	3,812	−0.63 to 0.65	3,177	−0.63 to 0.64
*z* Score FEV_1_/FVC ratio at 15 y†	3,657	−0.52 to 0.72	3,046	−0.51 to 0.72
*z* Score FEF_25-75_ (SDU) at 15 y†	3,812	−0.66 to 0.63	3,177	−0.67 to 0.64

*IQR*, Interquartile range.

∗ Number of participants with data available.

† Height-, age-, and sex-adjusted standard SDUs.

‡ BDR of FEV_1_ defined as a change of greater than 12% in FEV_1_.

§ Confounders: sex, parity, maternal history of asthma or allergy, maternal smoking and anxiety during pregnancy, and preterm delivery.

**Table IV:** Adjusted associations of wheezing phenotypes at 0 to 16½ years with doctor-diagnosed asthma ever (n = 5595), Feno values (n = 1721), and FEV_1_ reversibility (n = 3063) at 14 to 15 years in participants with at least 2 observations of wheeze

Wheezing phenotype at 0-16½ y	Doctor-diagnosed asthma ever at 14 y		Feno ≥35 ppb at 14-15 y		BDR >12% at 15 y	
	No. of asthmatic patients/total∗	Adjusted OR (95% CI)†	n_1_/total∗	Adjusted OR (95% CI)†	n_2_/total∗	Adjusted OR (95% CI)†
Never/infrequent wheeze	272/3661	1 (reference)	290/1157	1 (reference)	139/2003	1 (reference)
Preschool-onset remitting wheeze	173/870	2.73 (2.22-3.36)	61/243	1.00 (0.73-1.35)	33/472	1.18 (0.81-1.70)
Midchildhood-onset remitting wheeze	231/337	25.1 (19.4-32.4)	42/102	1.87 (1.23-2.84)	21/204	1.77 (1.11-2.82)
School age–onset persisting wheeze	187/223	50.0 (35.2-71.0)	57/82	6.24 (3.82-10.21)	27/127	3.34 (2.08-5.35)
Late childhood–onset persisting wheeze	161/250	19.8 (15.0-26.2)	38/66	3.70 (2.30-5.95)	19/131	2.14 (1.28-3.58)
Continuous wheeze	246/254	367.6 (181-747)	49/71	6.74 (3.93-11.54)	19/126	2.61 (1.53-4.45)

*n*_*1*_, Number of participants with Feno vales of 20 ppb or greater; *n*_*2*_, number of participants with BDR.

∗ Number of participants with data available (approximated from modal assignment).

† Adjusted for sex, parity, maternal history of asthma or allergy, maternal smoking and anxiety during pregnancy, preterm delivery, low birth weight, and day care attendance during the first year.

**Table V:** Adjusted association of wheezing phenotypes at 0 to 16½ years with lung function measures at 15 years (postsalbutamol *z* scores) in participants with at least 2 observations of wheeze and spirometry done at 15 years

Wheezing phenotype at 0-16½ y	FEV_1_ (SDU) at 15 y		FVC (SDU) at 15 y		FEV_1_/FVC ratio (SDU) at 15 y		FEF_25-75_ (SDU) at 15 y	
	No.∗	Adjusted mean difference (95% CI)†	No.∗	Adjusted mean difference (95% CI)†	No.∗	Adjusted mean difference (95% CI)†	No.∗	Adjusted mean difference (95% CI)†
Never/infrequent wheeze	1997	0 (reference)	2094	0 (reference)	1997	0 (reference)	2094	0 (reference)
Preschool-onset remitting wheeze	463	−0.03 (−0.12 to 0.07)	477	0.05 (−0.05 to 0.14)	463	−0.15 (−0.25 to −0.05)	477	−0.14 (−0.24 to −0.04)
Midchildhood-onset remitting wheeze	203	0.09 (−0.06 to 0.23)	209	0.20 (0.06 to 0.34)	203	−0.22 (−0.36 to −0.08)	209	−0.16 (−0.30 to −0.01)
School-age–onset persisting wheeze	127	0.08 (−0.10 to 0.26)	131	0.18 (0.00 to 0.35)	127	−0.22 (−0.39 to −0.04)	131	−0.16 (−0.33 to 0.02)
Late childhood–onset persisting wheeze	130	−0.02 (−0.19 to 0.15)	135	0.02 (−0.15 to 0.18)	130	−0.08 (−0.25 to 0.09)	135	−0.21 (−0.38 to −0.04)
Continuous wheeze	126	−0.08 (−0.27 to 0.10)	131	0.04 (−0.14 to 0.22)	126	−0.27 (−0.45 to −0.09)	131	−0.33 (−0.51 to −0.15)

∗ Number of participants with data available (approximated from modal assignment).

† Adjusted for sex, parity, maternal history of asthma or allergy, maternal smoking and anxiety during pregnancy, preterm delivery, low birth weight, and day care attendance during the first year.

**Table VI:** Strength and direction of associations between extended wheezing phenotypes and clinical outcomes at 14 to 15 years

Phenotype	Asthma	Feno ≥35 ppb	BDR	FEV_1_	FVC	FEV_1_/FVC ratio	FEF_25-75_
Preschool-onset remitting wheeze	√	×	×	×	×	↓	↓
Midchildhood-onset remitting wheeze	√√	√	√	×	↑↑	↓↓	↓
School-age–onset persisting wheeze	√√√	√√√	√	×	×	↓↓	×
Late childhood–onset persisting wheeze	√√	√√	√	×	×	×	↓↓
Continuous wheeze	√√√√	√√√	√	×	×	↓↓	↓↓

The strength of association of each wheezing phenotype with each outcome is represented by the number of symbols (√, ↓, and ↑), with × representing absence of association with that outcome.

## Methods

### Participants

ALSPAC recruited 14,541 pregnant women resident in Avon, United Kingdom, with expected dates of delivery from April 1, 1991, to December 31, 1992. This number (14,541) is the initial number of pregnancies for which the mother enrolled in the ALSPAC study and had either returned at least 1 questionnaire or attended a “Children in Focus” clinic by July 19, 1999. Of these initial pregnancies, there were 14,676 fetuses, resulting in 14,062 live births and 13,988 children who were alive at 1 year of age. When the oldest children were approximately 7 years of age, an attempt was made to bolster the initial sample with eligible cases who did not join the study originally. As a result, when considering variables collected from the age of 7 years onward (and potentially abstracted from obstetric notes), there are data available for more than the 14,541 pregnancies mentioned above. The number of new pregnancies not in the initial sample (known as phase I enrollment) that are currently represented on the built files and reflecting enrollment status at the age of 18 years is 706 (452 and 254 recruited during phases II and III, respectively), resulting in an additional 713 children being enrolled. The phases of enrollment are described in more detail in the cohort profile paper.^E1^ Therefore the total sample size for analyses using any data collected after the age of 7 years is 15,247 pregnancies, resulting in 15,458 fetuses. Of this total sample of 15,458 fetuses, 14,775 were live births, and 14,701 were alive at 1 year of age.

A 10% sample of the ALSPAC cohort, known as the Children in Focus group, attended clinics at the University of Bristol at various time intervals between 4 to 61 months of age. The Children in Focus group was chosen at random from the last 6 months of ALSPAC births (1432 families attended ≥1 clinic). Excluded were those mothers who had moved out of the area or were lost to follow-up and those partaking in another study of infant development in Avon.

Please note that the study Web site contains details of all data that are available through a fully searchable data dictionary and reference the following Web page:

http://www.bris.ac.uk/alspac/researchers/data-access/data-dictionary/.

### Skin prick tests

Atopy was measured at a research clinic at age 7½ years, as previously described.^E2^ A positive response was defined as a mean wheal diameter of greater than 2 mm with an absent response to negative control solution, and atopy was defined as a positive response to 1 or more of house dust mite, cat, or grass pollen.

### Feno at 14 to 15 years

Feno values were measured online at a constant flow of 50 mL/s according to European Respiratory Society and American Thoracic Society guidelines by using a Sievers NOA-280i nitric oxide analyzer (GE Analytical Instruments, Boulder, Colo).

Additionally, 758 children with less stringent criteria (individual exhalations >5% deviation but at least 1 within the acceptable range of 10-200 ppb) were included. Feno measurements were done before spirometric measurements. Children were requested to omit their inhaled corticosteroids, if applicable, 48 hours before their visit to the clinic. At the time of measurements, 14 (0.3%) children received oral steroids, 221 (5.2%) had a chest infection and/or fever with a cold in the preceding 3 weeks, and 39 (0.9%) and 89 (2.1%) had used short- or long-acting bronchodilators for 6 or 24 hours, respectively, before the respiratory assessments. These children and those with less stringent criteria of Feno values were included in our analyses because we observed no differences in results when they were included or excluded.

### Asthma status at 0-14 years

Using ever-reported doctor-diagnosed asthma at 91 and 166 months and maternal report of asthma at 166 months, we derived the following asthma status variable: *no asthma* if negative at both 91 and 166 months, *remittent asthma* if positive at 91 months but negative at 166 months or positive at both 91 and 166 months but negative maternal report of asthma in the last 12 months at 166 months, *incident asthma* if negative at 91 months but positive at 166 months, and *persistent asthma* if positive at both 91 and 166 months and positive maternal report of asthma in the last 12 months at 166 months.

### Potential confounders

We obtained details of maternal educational attainment (lower level defined as educated to school leaving certificate at ≤16 years of age), smoking history, and personal history of asthma or allergy from questionnaires sent to the mother during pregnancy. The child's sex was obtained from delivery health care records. Postnatal maternal questionnaires from 3 to 15 months after birth were used to ascertain environmental tobacco smoke exposure.

### Statistical analysis

Associations of wheezing phenotypes with late asthma outcomes (physician-diagnosed asthma and objective measurements of lung function and Feno values) were examined by using logistic or linear regression models weighted for the probability of each subject of belonging to each phenotype to minimize the risk of bias caused by misclassification. These probabilities were estimated previously and referred to as the posterior probabilities.^E3^ Crude and adjusted ORs (for binary outcomes) and mean differences (for continuous outcomes) were derived in relation to the never/infrequent wheezing phenotype (reference group). Adjustment was done for potential confounders: sex, maternal lower education level, having 1 or more siblings (parity), maternal history of asthma or allergy, maternal smoking during pregnancy, maternal anxiety during pregnancy (as being in the fourth quantile of the Crown-Crisp Experiential Index),^E4^ low birth weight (<2.5 kg), preterm delivery (<37 weeks), and day care attendance during the first year.

## Results

A total of 6,330 (51.5%) participants were male, 5,139 (46.2%) had an asthmatic or allergic mother, 2,934 (26.1%) were exposed to maternal smoking during pregnancy, and 6,148 (54.9%) had at least 1 sibling. Five hundred seventy-five (5.0%) had low birth weight, and 682 (5.8%) were born preterm. Two thousand five hundred twenty-nine (23.4%) were exposed to maternal smoking during the first year, and 641 (6.0%) attended day care during the first year. Two thousand eight hundred seventy-four (26.1%) participants had wheezing reported at 6 months: the proportion of participants with reported wheezing decreased to 1,756 (17.6%) by 3½ years, 1,050 (12.9%) by 8½ years, and 592 (10.5%) by 16½ years. Of 6,691 participants with data available on skin prick tests at 7½ years, 1,403 (21.0%) were atopic. Comparing the samples of children with complete (n = 3,170) and incomplete data (≥2 observations of wheeze, n = 9,133) on wheezing in Table E1, we observed that incomplete data are associated with markers of social deprivation: increased exposure to tobacco smoke, maternal anxiety, maternal lower education, parity, low birthweight, and preterm delivery in those subsequently lost to follow up. Also, the proportion of wheezing differs between 6 and 69 months but is similar thereafter, which is consistent with less wheezing reported among children with complete data compared with those subsequently lost to follow-up. Our analyses included children with incomplete data on wheeze, avoiding bias that could be introduced in complete case analyses.

Male sex (adjusted multinomial odds ratio [mOR] range, 1.32-1.80) was associated with a similar higher risk of each phenotype (heterogeneity *P* = .13, see Table E2). Maternal lower education level was associated with a higher risk of midchildhood-onset remitting wheeze (mOR, 1.22 [95% CI, 1.02-1.45] with little evidence of heterogeneity; *P* = .11). Having at least 1 sibling was associated with preschool- and midchildhood-onset remitting and continuous wheezing phenotypes, and there was little evidence for association with the other phenotypes (heterogeneity *P* = .0012). Maternal history of asthma or allergy was associated with a higher risk of each wheezing phenotype (mOR, 1.27-2.30, heterogeneity *P* < .0001), and the strongest associations were with midchildhood-onset remitting (mOR, 1.65 [95% CI, 1.40-1.93]) and continuous (mOR, 2.30 [95% CI, 1.88-2.81]) wheezing. Maternal smoking during pregnancy was associated with a higher risk of preschool- and midchildhood-onset remitting and continuous wheeze phenotypes (mOR, 1.33-1.46; heterogeneity *P* = .71). Prenatal maternal anxiety was associated with a higher risk of each wheezing phenotype (mOR, 1.12-1.29; heterogeneity *P* = .08). Low birth weight was associated with continuous wheeze (mOR, 1.63 [95% CI, 1.00-2.64]; heterogeneity *P* = .26). Preterm delivery was associated with higher risk of preschool- and midchildhood-onset remitting and continuous wheezing (mOR, 1.35-1.63; heterogeneity *P* = .30). Day care attendance during the first year was associated with a higher risk of preschool-onset remitting wheeze phenotype, although we found little evidence of heterogeneity (*P* = .16). We found little evidence of an association between maternal smoking during the first year and any of the wheezing phenotypes.
